# Mental health of healthcare workers during the first year of the COVID-19 pandemic in the Netherlands

**DOI:** 10.1093/eurpub/ckac129.175

**Published:** 2022-10-25

**Authors:** M van der Noordt, KI Proper, B Loef, CRL Boot, FM Kroese, M de Bruin, SH van Oostrom

**Affiliations:** 1 Center for Health and Society, RIVM, Bilthoven, Netherlands; 2 Center for Nutrition, RIVM, Bilthoven, Netherlands; 3 Department of Public and Occupational Health, Amsterdam UMC, VU University Amsterdam, Amsterdam, Netherlands

## Abstract

**Purpose:**

In March 2020, the WHO declared COVID-19 a pandemic. Previous virus outbreaks, such as the SARS outbreak in 2003, appeared to have a great impact on the mental health of healthcare workers. The aim of this paper is to study to what extent mental health of healthcare workers differed from non-healthcare workers during the first year of the COVID-19 pandemic.

**Methods:**

We used data from a large-scale longitudinal online survey conducted by the Corona Behavioral Unit in the Netherlands. Eleven measurement rounds were analyzed, from April 2020 to March 2021 (N = 16,657; number of observations=64,316). Mental health, as measured by the 5-item Mental Health Inventory, was compared between healthcare workers and non-healthcare workers over time, by performing linear GEE-analyses.

**Results:**

Mental health scores were higher among healthcare workers compared to non-healthcare workers during the first year of the pandemic (1.29 on a 0-100 scale; 95%-CI=0.75-1.84). During peak periods of the pandemic, with over 100 hospital admissions or over 25 ICU admissions per day and subsequently more restrictive measures, mental health scores were observed to be lower in both healthcare workers and non-healthcare workers.

**Conclusions:**

During the first year of the COVID-19 pandemic, we observed no relevant difference in mental health between healthcare workers and non-healthcare workers in the Netherlands. To be better prepared for another pandemic, future research should investigate which factors hinder and which factors support healthcare workers to maintain a good mental health.

**Key messages:**

• During the first year of the COVID-19 pandemic, we observed no relevant difference in mental health between healthcare workers and non-healthcare workers in the Netherlands.

• During peak periods of the pandemic, mental health was observed to be poorer in both healthcare workers and non-healthcare workers.

